# Blockade of ACK1/TNK2 To Squelch the Survival of Prostate Cancer Stem-like Cells

**DOI:** 10.1038/s41598-018-20172-z

**Published:** 2018-01-31

**Authors:** Nupam P. Mahajan, Domenico Coppola, Jongphil Kim, Harshani R. Lawrence, Nicholas J. Lawrence, Kiran Mahajan

**Affiliations:** 1 Drug Discovery Department, Moffitt Cancer Center, 12902 Magnolia Drive, Tampa, FL 33612 USA; 2 Department of Pathology, Moffitt Cancer Center, 12902 Magnolia Drive, Tampa, FL 33612 USA; 3 Biostatistics Department, Moffitt Cancer Center, 12902 Magnolia Drive, Tampa, FL 33612 USA; 4 Chemical Biology Core, Moffitt Cancer Center, 12902 Magnolia Drive, Tampa, FL 33612 USA; 5 Tumor Biology Department, Moffitt Cancer Center, 12902 Magnolia Drive, Tampa, FL 33612 USA; 6 Department of Oncologic Sciences, University of South Florida, Tampa, FL 33612 USA

## Abstract

Prostate cancer stem-like cells (PCSCs) are not only enriched in the CD44^+^PSA^−/lo^ subpopulation but also employ androgen-independent signaling mechanisms for survival. CD44^+^ PCSCs defy androgen deprivation, resist chemo- and radiotherapy and are highly tumorigenic. Human prostate tissue microarray (TMA) staining revealed an increased membranous staining of CD44 in the luminal compartment in higher grade G7-G9 tumors versus staining of the basal layer in benign hyperplasia. To uncover tyrosine kinase/s critical for the survival of the CD44^+^PSA^−/lo^ subpopulation, we performed an unbiased screen targeting 87 tyrosine kinases with gene specific siRNAs. Among a subset of tyrosine kinases crucial for PCSC survival, was a non-receptor tyrosine kinase, ACK1/TNK2, a critical regulator of castration resistant prostate cancer (CRPC) growth. Consistently, activated ACK1 as measured by phosphorylation at Tyr284 was significant in the CD44^+^PSA^−/lo^ population. Conversely, pharmacological inhibition by ACK1 inhibitor, (*R*)-**9b**MS mitigated CD44^+^PSA^−/lo^ sphere formation, overcame resistance to radiation-induced cell death, induced significant apoptosis in PCSCs and inhibited CD44^+^PSA^−/lo^ xenograft tumor growth in castrated mice suggesting dependency of PCSCs on ACK1 for survival. Thus, blockade of ACK1/TNK2 could be a new therapeutic modality to target recalcitrant PCSCs.

## Introduction

Prostate Cancer (PC) is a commonly diagnosed cancer with 233,000 new cases each year and has emerged to be a significant cause of cancer related deaths among American men^1^. While early stage PCs often responds to surgery or radiotherapy, two commonly employed therapeutic interventions; the advanced prostate cancers are generally linked to poor prognosis^1,2^. Advanced disease is treated either with Androgen Deprivation Therapy (ADT) that includes surgical or chemical castration, anti-androgens (both first and second generation androgen receptor antagonists) and/or radiotherapy^2,3^. However, the disease invariably recurs. The molecular mechanisms by which PC cells evade treatment appear to be varied with the cells activating alternative survival pathways in response to specific treatment/s and thus present a formidable challenge for PC therapy^2,4–8^. Resistance is often linked to a reiterative feed forward signaling mechanism driving tumor heterogeneity that allow subsets of cancer cells in the population to escape death.

Cancers are presumed to be driven by cancer stem cells (CSC), or tumor-initiating cells, which often constitute <0.1% fraction of a cancer cell population^9,10^. These cells with undifferentiated stem cell-like properties coexist within a niche comprised of a heterogeneous tumor mass and a favorable tumor microenvironment^10,11^. CSCs in many solid tumors, including cancers of the prostate, breast, pancreas, head and neck, and ovary have been identified using the cell surface adhesion molecule CD44, either individually or in combination with other cell surface markers such as CD133, integrin and ALDH1A1^9,12^. CD44 is a multifunctional receptor, expressed in many cancer cells, regulates inter cellular communication, communication with extracellular matrix, homing to lymph nodes and promotes metastasis^13^. Prostate Cancer Stem Cell-like population (PCSCs) with enhanced clonogenic, tumor-initiating and metastatic capacities are not only enriched for CD44 expression, but may also harbor low levels of androgen-dependent AR target gene expression such as PSA due to their ability to generate a mixed population of AR positive and AR low or negative cells in castration resistant tumors^9,14–16^, suggesting that this CD44^+^PSA^−/lo^ subpopulation employs androgen-independent signaling mechanisms for survival^17,18^. Interestingly, these PCSCs (CD44^+^PSA^−/lo^ population) not only possess a significant capacity to reinitiate serially transplantable tumors in mice, but also often overcome ADT and chemotherapy^14^.

One mechanism that has been suggested for cancer recurrence or relapse is the activation of growth factor stimulated non-receptor tyrosine kinase (TK) regulated signaling pathways that can bypass androgen requirements of PC cells^3,19^. ACK1 has been shown to be critical for progression of PC to hormone therapy insensitive stage called castration resistant prostate cancer or CRPC due to it’s ability to regulate the expression and function of AR in androgen-independent manner^8,20–25^. However, whether ACK1 has role in PCSC survival is not known. In this study, we report identification of ACK1 as a critical regulator of PCSC survival.

## Results and Discussion

### Alteration in CD44 expression during prostate tumor progression

**Figure 1:**
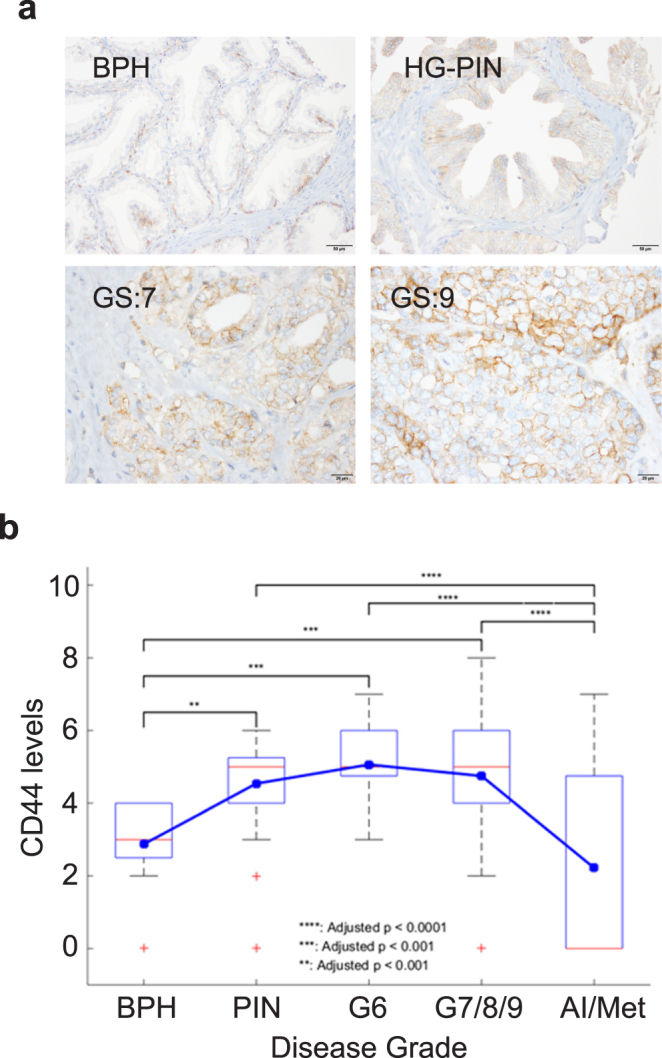
An increased CD44 staining in the luminal compartment of higher Gleason grade G7-G9 prostate tumors. **(a)** TMA sections representing different prostate cancer grades stained with anti-CD44 antibodies. **(b)** Box plots to summarize distributions of CD44 staining intensities in different grades of prostate cancer. The box has lines at the lower quartile (25%), median (50%), and upper quartile values (75%), whereas the blue circle marks the mean value. Whiskers extend from each end of the box to the most extreme values within 1.5 times the interquartile range from the ends of the box. The data with values beyond the ends of the whiskers, displayed with red crosses, are potential outliers.

Albeit rare, PCSCs are reported to be present in a number of PC cell lines and primary prostate tumors^9,14^. To evaluate the expression of CD44 during progression of the disease we performed tumor microarray analysis (TMA) of human prostate biopsies representing various grades of the disease. Immunohistochemical (IHC) staining of the TMA revealed variable membranous staining of CD44. An increased membranous staining of CD44 was observed in the luminal compartment in higher Gleason grade G7-G9 tumors versus staining of the basal layer in benign prostatic hyperplasia (BPH) (Fig. 1a). The expression of CD44 in BPH was observed to be significantly lower than prostatic intraepithelial neoplasia (PIN), G6, and G7/8/9/10. Intriguingly, the expression of CD44 was also significantly lower in androgen independent/metastatic PCs (AI/Meta) when compared to PIN, G6, and G7/8/9/10. There was no difference between AI/Meta and BPH as well as no difference between PIN, G6, and G7/8/9/10. Notably, the G6 stage displayed high CD44 levels (p < 0.0001) compared to other stages (Supplementary Table S1; Fig. 1b).

### Screening of the human tyrosine kinome to identify druggable targets essential for PCSC survival

**Figure 2:**
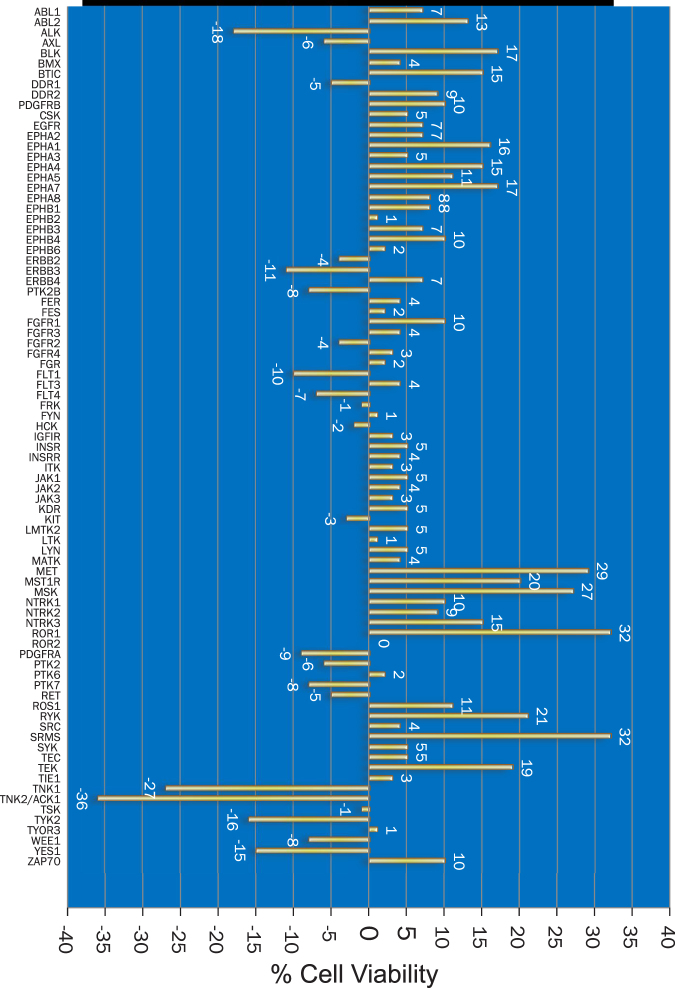
ACK1/TNK2 ablation compromises the viability of CD44^+^PSA^−/lo^ PCSCs. Graphical representation showing percent viability of the CD44^+^PSA^−/lo^ PCSCs following depletion of various tyrosine kinases.

We enriched CD44^+^ population from the androgen dependent LNCaP PC cell line by staining with anti-CD44-PE antibodies followed by fluorescence activated cell sorting (FACS) (Supplementary Figure S1a). We also examined the expression of PSA in the CD44-enriched population. Compared to the parental population, the PSA expression was significantly reduced in the CD44^+^ population (Supplementary Figure S1b). To determine whether tyrosine kinases regulate PCSC survival, an unbiased screen was designed wherein CD44^+^PSA^−/lo^ PCSCs isolated from LNCaP cells were transfected with siRNA library consisting of 3 sets of siRNA for each of the 85 distinct receptor and non-receptor tyrosine kinases (Supplementary Figure S1c). After 48 hours, cells were harvested in culture media, stained with CD44-PE monoclonal antibody and DAPI stain to distinguish live/dead population and live cells were analyzed by flow cytometry. Depletion of ACK1/TNK2, TNK1, TYK2, YES1 and ALK kinases caused the most decrease in the number of CD44^+^ cells, compared to scrambled siRNA. Importantly, silencing of ACK1/TNK2 caused the most reduction in the number of live CD44^+^PSA^−/lo^ cells (Fig. 2, Supplementary Table S2).

Bioinformatics analysis was performed to assess whether the top five ‘hits’ are altered in human prostate cancers (cBioPortal Database). It revealed that the 5 kinase gene set (ACK1, TNK1, TYK2, YES1 and ALK) expression is altered in 39 of the 85 (45.9%) queried samples. Interestingly, these constituted 5 distinct nodal points in STRING network analysis (Supplementary Figure 2a). ACK1 and TYK2 gene expression was modulated in 29% of prostate cancers^26^ (Supplementary Figure 2b). Moreover, ACK1 exhibited significant tendency towards co-occurrence with TYK2 (p < 0.001; Log Odds Ratio 2.113). In contrast, other kinases (TNK1, YES1 and ALK) exhibited a low tendency towards co-occurrence or tendency towards mutual exclusivity (Supplementary Figure 2c). Taken together these data underscore an important role for ACK1 in PCSC survival.

### Sphere formation and radio-resistance of CD44^+^PSA^−/lo^ PCSCs can be mitigated by ACK1 inhibition

**Figure 3:**
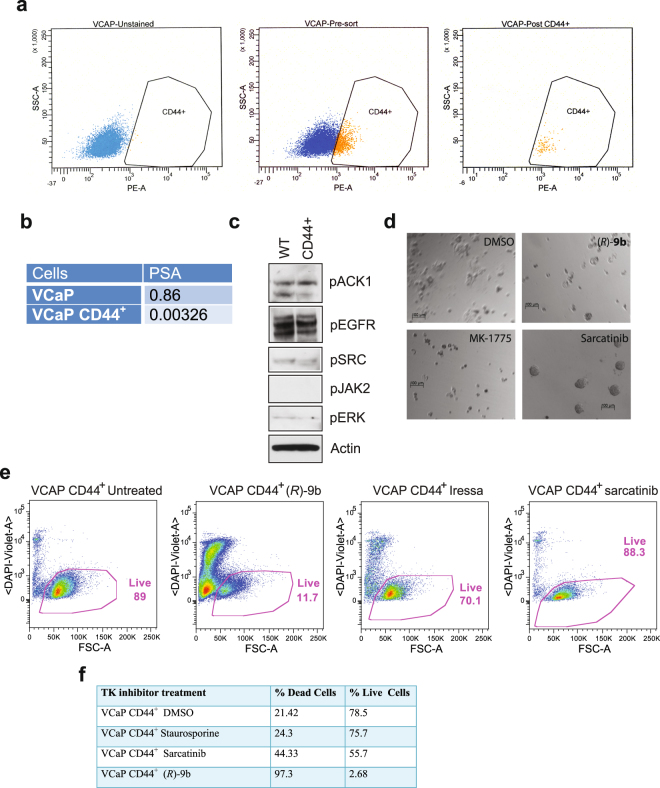
ACK1 inhibitor causes death of prostate cancer stem-like cells (PCSCs). **(a)** CD44^+^ VCaP cells were isolated by sorting cells following staining with the CD44-PE antibodies. (**b**) Relative levels of PSA determined by qRT-PCR. (**c**) Analysis of phosphorylation status of tyrosine kinases in the CD44^+^PSA^−/lo^ VCaP population. Whole cell extracts made from WT and CD44^+^PSA^−/lo^ cells were analyzed by immunoblotting with specific antibodies. (**d**) CD44^+^PSA^−/lo^ PCSCs were grown for 2–3 days as single cells on a very thin film of matrigel coated plates and treated with (*R*)-**9b**, Sarcatinib or AZD-1775 and images were captured after colony formation. **(e)** CD44^+^ PCSCs obtained from VCaP were treated overnight with 5 uM of (*R*)-**9b**, Sarcatinib or Iressa. Cells were CD44 or DAPI stained followed by FACS analysis. **(f)** PCSCs obtained from VCaP were treated overnight with 10 uM of (*R*)-**9b**, Sarcatinib or staurosporine (1000 nM). Caspase-3 and Sytox staining was performed followed by FACS analysis. Percent live/dead cells were determined.

To confirm ACK1 regulates the survival of the PCSCs, CD44^+^PSA^−/lo^ were first enriched from VCaP cells by staining with anti-CD44 antibodies, followed by FACS analysis. 86% of the VCaP cells were positive for this stem cell marker after FACS analysis (Fig. 3a). Quantitative profiling revealed low PSA levels in the enriched CD44^+^ population compared to parental cells (Fig. 3b). Subsequently, protein extracts were prepared from WT and CD44^+^PSA^−/lo^ cells and immunoblotting was performed. Phosphorylation of ACK1 at Tyr284 site was detected in both WT and sorted PCSC CD44^+^ populations suggesting a requirement for ACK1 function in both cell types (Fig. 3c).

CSCs form spheroids characteristic of undifferentiated cells on non-adherent surface. To assess the effect of ACK1 inhibition on sphere formation, CD44^+^ PSA^−/lo^ PCSCs from VCaP cells were grown for 2–3 days as single cells on matrigel coated plates. Subsequently, cells were treated with 5 uM of ACK1-specific small molecule inhibitor, (*R*)-**9b**^25,27^. As a control, cells were also treated with SRC inhibitor (Sarcatinib), WEE1 inhibitor (AZD-1775, also known as MK-1775) and sphere formation was determined 2 weeks later. Both, ACK1 and WEE1 inhibitors suppressed sphere formation. In contrast, robust sphere formation was detected in PCSC cells treated with SRC inhibitor, suggesting ACK1 as a critical survival kinase in PCSCs (Fig. 3d).

As a further confirmation of the requirement of ACK1 in PCSC survival, the PCSCs (CD44^+^ PSA^−/lo^ population) were treated with (*R*)-**9b**, Sarcatinib or the EGFR inhibitor, Iressa. 24 hours later the percentage of live cells (DAPI negative) were determined by flow cytometry. PCSCs were observed to be highly refractory to Sarcatinib and Iressa treatments and exhibited 88% and 70% live cells, respectively, compared to (*R*)**9b** which exhibited 11% of live cells (Fig. 3e). To further confirm this observation, we performed caspase cell death assay and observed that PCSCs were extremely sensitive to the ACK1 inhibitor (*R*)-**9b**; only 2.68% PCSCs survived the (*R*)-**9b** treatment, in agreement with the detection of activated ACK1 in this population (Fig. 3f).

### CD44 enriched PCSC cells are dependent on ACK1 for formation of xenograft tumors

**Figure 4:**
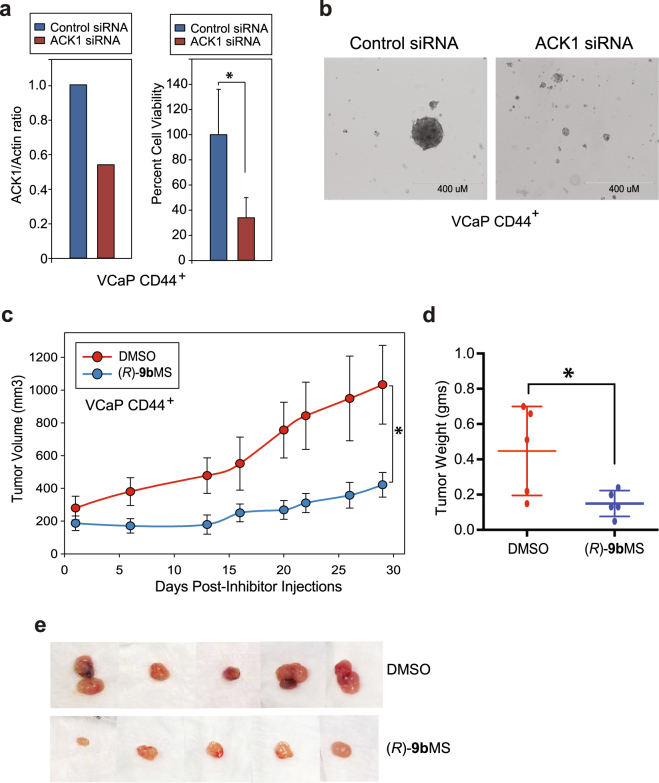
Genetic or pharmacological ablation of ACK1 suppresses proliferation, reduces sphere formation and inhibits xenograft tumor growth. **(a)** VCaP CD44^+^PSA^−/lo^ cells were transfected with ACK1 or control siRNAs. Quantitative RT-PCR was performed to confirm knockdown of ACK1 with ACK1 specific siRNAs. Actin was used as the normalization control. Results are representative of two independent transfection experiments (n = 3; technical replicates) (left panel). Viability of the VCaP CD44^+^PSA^−/lo^ cells was examined 72 hours after transfection. The number of viable cells were determined by trypan blue exclusion assay (n = 2, 3 replicates) (right panel). **(b)** CD44^+^PSA^−/lo^ VCaP cells were transfected with ACK1 or control siRNAs and sphere formation was examined. Scale bar is 400 uM. **(c)** CD44^+^ VCaP cells were injected in castrated SCID mice, and after palpable tumor formation, mice were castrated and then injected with DMSO or (*R*)-**9b**MS. Mean tumor volume is shown post inhibitor injection. *p < 0.05. **(d)** Xenograft tumors were excised and shown. *p = 0.034. **(e)** Xenograft tumors were excised and weighed, the individual tumor weights are shown. **(e)** Xenograft tumors were excised and weighed, the individual tumor weights are shown.

To determine that (*R*)-**9b** mediated inhibition of proliferation of CD44^+^PSA^−/lo^ cells isolated from VCaP cells is not due to the ‘off target’ effect, we ablated ACK1 using siRNAs, followed by cell proliferation and sphere formation assay. ACK1 knockdown resulted in a significant inhibition of proliferation of CD44^+^PSA^−/lo^ cells, which was also reflected in compromised sphere formation (Fig. 4a and b).

Our recent studies reveal that (*R*)-**9b**MS is a potent inhibitor of CRPC growth^25^. To examine the tumor forming proficiency of the PCSCs, we isolated CD44^+^ cells from VCaP. VCaP cells are androgen dependent and form xenograft tumors in intact mice. However, if mice are castrated following initial tumor establishment, the tumor growth is not impaired^28^. In brief, SCID mice were injected with CD44^+^PSA^−/lo^ VCaP cells and once mice formed palpable tumors in (about 5–6 weeks), these mice were injected with (*R*)-**9b**MS (25 mg/Kg of body weight) or DMSO (Vehicle) as a control. Mice were injected twice a week for 4 weeks and xenograft tumors were measured. In contrast to the vehicle treated mice, (*R*)-**9b**MS injected mice exhibited significant suppression of xenograft tumor growth (Fig. 4c and d).

The mice were humanely euthanized at the end of the experiment and tumors were excised and weighed. The weights of the tumors reveal that (*R*)-**9b**MS treatment significantly inhibited xenograft tumor growth (Fig. 4d). The tumors in mice that were injected with that (*R*)-**9b**MS were very pale in color, very likely to be due to significant loss of neo-angiogenesis upon ACK1 inhibition (Fig. 4e).

### Enhanced radiosensitization of PCSCs treated with the ACK1 inhibitor

**Figure 5:**
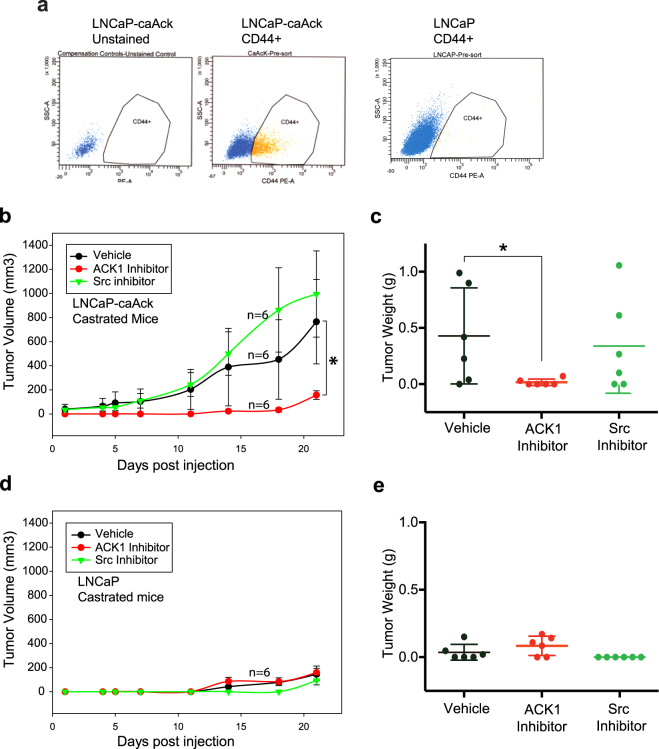
Xenograft tumor growth inhibition by ACK1 kinase inhibitor. **(a**) PCSCs from LNCaP-caAck or LNCaP were enriched by staining with anti-CD44 PE antibodies and sorted by flow cytometry. **(b)** LNCaP-caAck cells were injected in castrated SCID mice, and after palpable tumor formation, mice were injected with ACK1 or SRC inhibitor. Mean tumor volume is shown post inhibitor injection. *p < 0.05. **(c)** Xenograft tumors were excised and weighed, the individual tumor weights are shown. *p = 0.039. **(d)** LNCaP cells were injected in castrated SCID mice, and after about 4 weeks, mice were injected with ACK1 or SRC inhibitor. Mean tumor volume is shown post inhibitor injection. **(e)** Xenograft tumors were excised and weighed, the individual tumor weights are shown.

To assess the potential role of ACK1 inhibitor in overcoming radioresistance of PCSCs, PCSCs isolated from LNCaP cells stably expressing constitutively active ACK1 kinase (LNCaP-caACK cells)^20^ (Fig. 5a) were irradiated with ionizing radiation (IR) followed by treatment with (*R*)-**9b**. The (*R*)-**9b** treatment significantly sensitized PCSCs to radiation (Supplementary Figure S3, compare top row with bottom row).

### Targeting CD44+ PCSCs with ACK1 inhibitors

We observed that LNCaP-caACK, displayed higher levels of CD44 expression (~16% compared <1%) compared to the parental LNCaP cells (Fig. 5a). To examine the tumor forming proficiency of the two populations, castrated male nude mice (n = 6) were injected with LNCaP-caACK cells^20^. Once mice formed palpable tumors (about 4 weeks), these immunocompromised mice were injected with the first generation, ACK1 small molecule inhibitor, AIM-100^21,23^ (25 mg/kg of body weight), Sarcatinib (25 mg/kg of body weight) or DMSO (Vehicle) as a control. Mice were injected twice a week for 3 weeks and xenograft tumor growth was measured. LNCaP-caACK formed robust xenograft tumors and reached 1000 mm^3^ in 3 weeks in the vehicle treated group. In contrast, a significant suppression of xenograft tumor growth was seen in ACK1 inhibitor injected mice (Fig. 5b). However, treatment with the SRC inhibitor, Sarcatinib, did not mitigate tumor growth in a majority of the mice (Fig. 5b).

The mice were humanely euthanized at the end of the protocol, tumors were excised and weighed. The weights of the tumors revealed that ACK1 inhibitor treatment completely inhibited xenograft tumor growth, while Sarcatinib had no effect on majority of the tumors consistent with the tumor volumes (Fig. 5c). As a negative control, we injected parental LNCaP cells in castrated male nude mice to measure their tumor forming proficiency. LNCaPs are androgen-dependent cells and expectedly these cells did not form appreciable tumors in the castrated male nude mice (Fig. 5d and e).

Identification of prostate cancer cells with stem like properties- i.e. self-renewal, *in vivo* tumorigenecity, with an ability to recapitulate tumor heterogeneity and resistance to standard therapies has been a challenge. Previous studies have revealed that human xenograft derived CD44^+^ populations are highly enriched in tumorigenic and metastatic progenitor cells and CD44 is a key regulator of the stem like properties^15,29^. A subset of these CD44^+^ express low levels of prostate specific antigen PSA (PSA^−/lo^ PC) and are resistant to castration^9^. We have identified a CD44^+^PSA^−/lo^ PC subpopulation (PCSCs) in some of the well characterized PC cell lines (LNCaP, VCaP, LAPC4) that possess many stem-like characteristics. These data allowed us to assess whether non-receptor tyrosine kinases (NRTKs), regulators of androgen-independent AR activity^20,25^, have a role in PCSC survival.

A pathogenic role for NRTKs such as ACK1/TNK2 and SRC is particularly evident as both of these kinases can directly interact with the AR, to regulate ligand independent AR transcriptional activity and to promote prostate tumorigenesis^20,30,31^. These molecular mechanisms have been used as an underlying rationale for the use of SRC inhibitors in the treatment of prostate cancer^3,32^. Although highly sensitive to ACK1 inhibition, interestingly, our studies reveal that PCSCs are refractory to genetic and pharmacological blockade of SRC (Figs 2 and 3d). Consistently, Sarcatinib accelerated prostate tumor growth in LNCaP-caAck animal models of hormone refractory cancer (Fig. 5). These studies are highly significant and clinically relevant to understand the emergence of drug resistance; addition of Dasatinib to Docetaxel, a cytotoxic chemotherapy drug targeting microtubules, did not improve the overall survival in Phase 3 trials for chemotherapy-naive men with metastatic CRPC^33^. Further, in a phase 2 trial, toxicity was high in patients treated with Dasatinib (70 mg/kg twice daily) after chemotherapy and tolerability was poor with limited activity in CRPCs^3,34^. Similarly Sarcatinib was clinically ineffective as a monotherapy^35^. Recently, our studies of LAPC4 cells that were deprived of androgen for 10 days revealed a significant increase in ACK1 levels^25^. Activated ACK1 (pY284-ACK1) expression was readily detected in the CD44^+^PSA^−/lo^ PCSC population (Fig. 3c). Taken together, these data may explain why SRC inhibitors have not been successful in clinical setting- as it may have failed to eliminate the tumorigenic and recalcitrant CD44^+^PSA^−/lo^ PCSC population that appear to rely on ACK1 for survival.

Our data reveals ACK1 as a critical tyrosine kinase regulating survival of the CD44^+^PSA^−/lo^ PCSCs. Our earlier studies demonstrated that Activated ACK1 expression correlates with prostate cancer progression to castration resistance^20^, which includes CRPC patients treated with radiotherapy^23^. We observed that ACK1 is an excellent therapeutic target to inhibit resurgence of the chemo-resistant population. Indeed, treatment with ACK1 small molecule inhibitors induced apoptosis of the CD44^+^PSA^−/lo^ and mitigated tumor formation in castrated mice, consistent with its role as a therapeutic target. Overall, our studies reveal ACK1/TNK2 as a new therapeutic vulnerability in PCSCs.

## Methods

### Cell lines, Antibodies and Inhibitors

VCaP and LNCAP cell lines were obtained from ATCC. LNCaP-CaAck cells were developed, as described earlier^36^. ACK1 monoclonal Ab (A11), actin, phosphotyrosine and AR monoclonal antibodies were purchased from Santacruz; Anti-phospho-ACK1 (Tyr284, Upstate) were purchased from Cell Signaling. Anti-CD44-PE antibodies were purchased from BD Biosciences. (*R*)-**9b**MS and AIM-100 were synthesized at Moffitt Cancer Center as described earlier^27^. Control and ACK1 siRNAs were generated by custom synthesis (Qiagen) and the sequences have been described previously^20^. For immunoprecipitations, cells were lysed in receptor lysis buffer (RLB) containing 25 mmol/L Tris (pH 7.5), 500 mmol/L NaCl, 1% Triton X-100, 10% glycerol, phosphatase inhibitors (10 mmol/L NaF, 1 mmol/L Na_2_VO_4_), and protease inhibitor mix (Roche).

### Screen of the Tyrosine kinome with silencing RNAs

Predesigned kinase-specific siRNA library was obtained from Bioneer (Cat#SHS-0110-7). Cells were transfected with siRNA library consisting of 3 sets of siRNA for each of the 85 distinct receptor and non-receptor tyrosine kinases. In brief, siRNAs were transfected using X-tremeGENE siRNA transfection reagent (Roche; Cat.No.04476093001) in 6 well plates. The day before transfection, 3.0 × 10^5^ CD44^+^PSA^−/lo^ cells were plated in each well with 2.5 ml media to reach 50–60% confluency at the time of transfection. The growth medium was removed from the 6-well plate before transfection. This was followed by the addition of 900 μl fresh growth medium in each well. For each well to be transfected, the siRNA duplexes were prepared. The siRNA duplex were diluted in 100 μl growth medium without serum. This solution was incubated for 15 minutes at room temperature. The mixture was added to each well containing CD44^+^PSA^−/lo^ cells, which resulted in 1 ml as total volume. The cells were incubated for 48 hours at 37 °C in a CO_2_ incubator.

### Tissue Microarray (TMA) analysis of human prostate cancers

The human prostate TMA in this study was developed after obtaining exemption from IRB approval from University of South Florida (USF), Division of Research Integrity and Compliance. We used the prostate TMA for our study for which we are exempt from IRB approval (once again written exemption) for this study, as no personal information about patients is sought. All experiments and methods were performed according to the relevant guidelines and regulations of the IRB, University of South Florida (USF).

For assessment of CD44 expression levels in human prostate cancer, immunohistochemistry was carried out on high-density TMAs (*n* = 250 cores) containing biopsies from different grades of the disease as described previously^21,23,37^. For statistical assessment of CD44 expression in prostate cancer, box plots were used to summarize the intensity of sampling distribution among different grades. The Mantel-Haenszel Chi-square test was used to access the relationship between CD44 levels and grades of prostate cancer. The progression from benign prostatic hyperplasia to PINs, G6, G7, G8–10, and CRPC were used for the correlation analysis. Analysis of variance (ANOVA) was performed to examine whether the CD44 expression levels differ among different grades of disease. The Tukey-Kramer method was further performed to detect changes in CD44 expression levels between pairs of progression steps. This post hoc procedure adjusts for pairwise comparisons and simultaneous inference.

### Mice Xenograft Studies

Mice breeding and colony maintenance was performed according to Institutional Animal Care and Use Committee protocols approved by University of South Florida, Division of Research Integrity and Compliance. 1 × 10^6^ LNCaP-caAck or LNCaP cells were suspended in 100 μl of PBS and 100 μl of Matrigel (Discovery Labware, Bedford, MA) and injected subcutaneously into the flanks of male nude castrated mice. After about 4 weeks when mice formed palpable tumors of ~150 mm^3^, the mice were randomly divided into 3 different groups. Mice were injected with AIM-100 (25 mg/kg of body weight per injection), or Sarcatinib (25 mg/kg of body weight per injection) or DMSO (*n* = 6  mice for each treatment). Mice were injected twice a week for 3 weeks and xenograft tumor growth was measured twice weekly using calipers. The mice were humanely euthanized at the end of the experiment and tumors were excised and weighed.

CD44 enriched VCaP (1 × 10^6^) cells/ml were suspended in 100 μl of PBS and 100 μl of matrigel (Discovery Labware, Bedford, MA) and injected subcutaneously into the flanks of male SCID mice. After mice formed palpable tumors ~150 mm^3^, the mice were castrated and randomly divided into 2 different groups. Two weeks later, mice were injected with (*R*)-**9b**MS (25 mg/kg of body weight per injection) or DMSO (*n* = 5 /group). Mice were injected twice a week for 4 weeks and xenograft tumor growth was measured twice weekly using calipers. The mice were humanely euthanized at the end of the experiment and tumors were excised and weighed.

### Flow Cytometry analysis and enrichment of CD44 positive prostate cancer cells

5 × 10^5^ prostate cancer cells were aliquoted into assay tubes. 2–3 ml of incubation buffer (0.5% BSA in 1X phosphate buffered saline) was added to each tube and rinsed by centrifugation. The cells were resuspended in 90 ul of incubation buffer per assay tube and blocked in this buffer for 10 minutes at room temperature. 10 ul of CD44-PE conjugated antibody was added to the assay tubes and incubated for 30–60 minutes in the dark at room temperature. Excess unbound antibody was rinsed off by washing with the incubation buffer followed by centrifugation. The cells were resuspended in 0.5 ml PBS and acquired on FACS canto and analyzed by the FlowJo software. For purification and collection of the CD44 population cell sorting was performed on the Becton Dickinson FACSVantage Cell Sorter.

## Electronic supplementary material

Supplementary Information
