# Supplementary material for: Blockade of ACK1/TNK2 To Squelch the Survival of Prostate Cancer Stem-like Cells
Source: Sci Rep. 2018 Jan 31;8:1954. doi: 10.1038/s41598-018-20172-z (PMC5792546; doi:10.1038/s41598-018-20172-z)
Supplement: Supplementary file 1 — Supplementary Information [file 41598_2018_20172_MOESM1_ESM.pdf]

Supplementary Information

Blockade of ACK1/TNK2 To Squelch the Survival of Prostate Cancer Stem-like Cells

Nupam P. Mahajan[1,6]*, Domenico Coppola[2,5], Jongphil Kim[3,6], Harshani Lawrence[4,6] and Nicholas Lawrence[1,6] and Kiran Mahajan[5,6]*

[1]Drug Discovery Department, [2]Department of Pathology, [3]Biostatistics Department, [4]Chemical Biology Core, and [5]Tumor Biology Department

Moffitt Cancer Center

12902 Magnolia Drive, Tampa, FL 33612, USA

[6]Department of Oncologic Sciences,

University of South Florida, Tampa, FL 33612, USA

Supplementary Figures: 3

Supplementary Tables: 2

SUPPLEMENTARY FIGURE LEGENDS

Supplementary Figure S1. RNAi screen of the tyrosine kinome to identify kinases essential for $CD44^{+}PSA^{-/lo}$ PCSCs survival.

(a) $CD44^{+}$ LNCaP cells were isolated by sorting cells following staining with the CD44-PE antibodies, followed by fluorescence activated cell sorting (FACS). (**b**) Relative levels of PSA was determined by qRT-PCR. (**c**) Flow chart work-flow: $CD44^{+}PSA^{-/lo}$ PCSCs isolated from LNCaP were transfected with an siRNA library consisting of 3 sets of siRNAs for each of the 85 distinct receptor and non-receptor tyrosine kinases. After 48 hours, cells were harvested in culture media, stained with CD44-PE monoclonal antibody and DAPI to distinguish live/dead population and live cells were analyzed by flow cytometry.

Supplementary Figure S2. Network analysis of the 5 non-receptor tyrosine kinases identified in the siRNA screen to affect PCSCs survival.

(a) 5 non-receptor tyrosine kinases identified in the siRNA (TNK2, TNK1, ALK, YES and TYK1) represent distinct nodal points in network analysis **(b).** Bioinformatics analysis revealed a 3 kinase (ACK1/TNK2, TYK2 and YES1) Gene Set expression is altered in 39 of the 85 (45.9%) queried samples (TCGA). **(c).** ACK1/TNK2 exhibited significant tendency towards co-occurrence with TYK2 ($p<0.001$; Log Odds Ratio 2.113). In contrast, other kinases exhibited low tendency towards co-occurrence or tendency towards mutual exclusivity (c).

Supplementary Figure S3. ACK1 inhibitor (*R*)-**9b**MS **inhibits radioresistant PCSC colony formation.**

PCSCs were seeded in 6-well plate and irradiated at doses shown, followed by treatment with (*R*)-**9b**MS. After 10 days, the surviving cells were stained with crystal violet.

Supplementary Figure S1

a

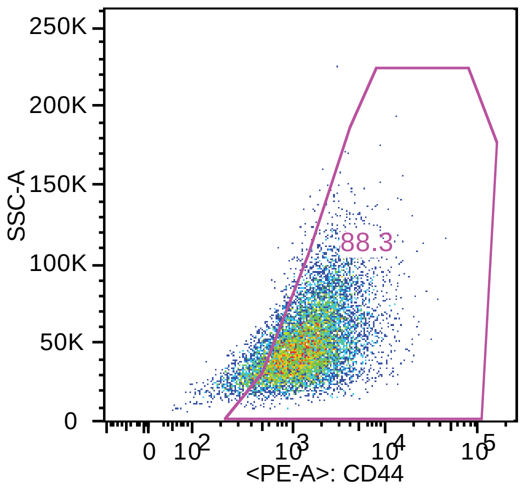

b

	Relative PSA levels
LNCaP	0.76
LNCaP CD44$^+$	3.7x10-^{5}

c

RNAi Screen of TK Kinome

Isolate the CD44 $^{+ve}$ PSA ^{lo} cancer stem cells

↓ PCSCs/LNCaP (0.5%-1%)

Minimally expand the CD44 population (2-3 weeks)

↓ Reconfirm CD44 expression by FLOW (~95%)

qRT-PCR to analyze AR, PSA, Oct-4, Nanog expression

↓ CD44 $^{+ve}$ AR^{lo}/PSA ^{lo} PCSCs express Oct4, Nanog

Transfect with an siRNA library (3 validated siRNAs against each target)

↓ Proof of Concept: Started with library against TKs (84)

Flow Cytometry analysis

↓ CD44 expression and Live/Dead

Validate the targets by complementary methods

Supplementary Figure S2

a

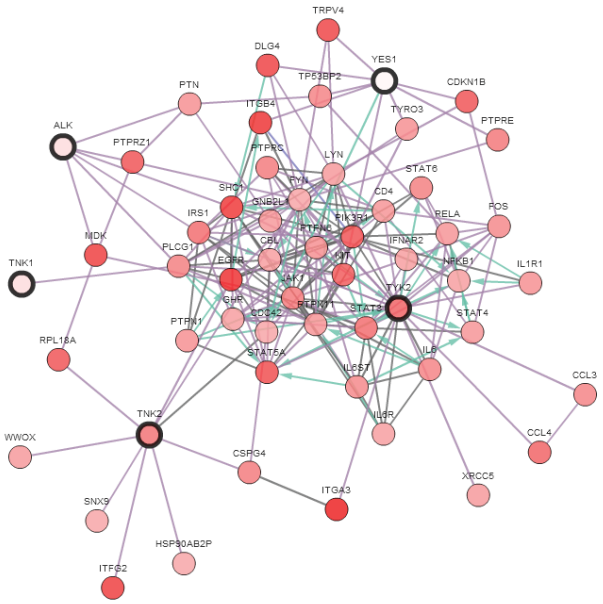

b

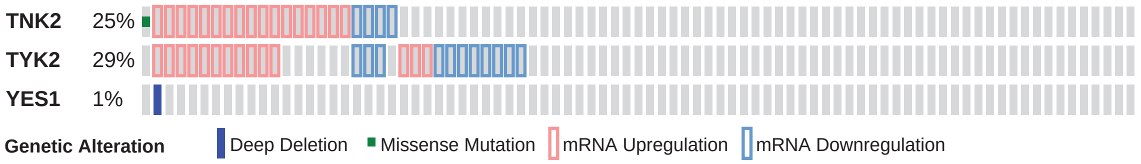

c

Gene A	Gene B	p-Value	Log Odds Ratio	Association
TNK2	TYK2	**<0.001**	2.113	Tendency towards co-occurrence
TNK1	TNK2	0.107	1.572	Tendency towards co-occurrence
ALK	TYK2	0.167	<-3	Tendency towards mutual exclusivity
TNK2	ALK	0.214	<-3	Tendency towards mutual exclusivity
TNK2	YES1	0.259	>3	Tendency towards co-occurrence
TNK1	ALK	0.267	1.558	Tendency towards co-occurrence
YES1	TYK2	0.294	>3	Tendency towards co-occurrence
TNK1	TYK2	0.462	0.502	Tendency towards co-occurrence
TNK1	YES1	0.941	<-3	Tendency towards mutual exclusivity
YES1	ALK	0.941	<-3	Tendency towards mutual exclusivity

Supplementay Figure S3

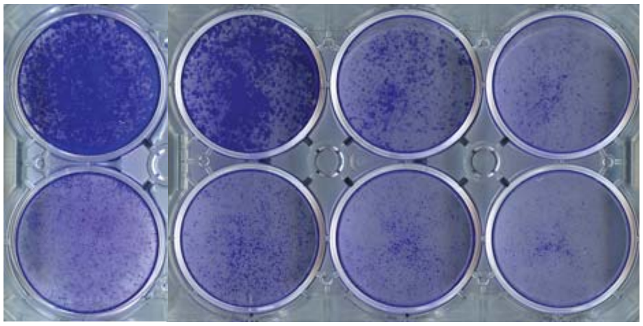

Table S1. Prostate TMA Data Analysis; CD44 expression by Disease Grade. Linear Association: no linear association between them observed (p-value by Mantel-Haenszel Chi-square test = 0.92).

CD44 level	Disease Grade										Total
	BPH		PIN		G6		G7/8/9/10		AI/Meta		
0	2	(8.3%)	2	(5.4%)	0	(0.0%)	15	(11.8%)	15	(55.6%)	34
2	4	(16.7%)	1	(2.7%)	0	(0.0%)	1	(0.8%)	0	(0.0%)	6
3	11	(45.8%)	1	(2.7%)	5	(15.2%)	10	(7.9%)	1	(3.7%)	28
4	7	(29.2%)	11	(29.7%)	3	(9.1%)	15	(11.8%)	4	(14.8%)	40
5	0	(0.0%)	13	(35.1%)	11	(33.3%)	28	(22.0%)	3	(11.1%)	55
6	0	(0.0%)	9	(24.3%)	13	(39.4%)	40	(31.5%)	2	(7.4%)	64
7	0	(0.0%)	0	(0.0%)	1	(3.0%)	13	(10.2%)	2	(7.4%)	16
8	0	(0.0%)	0	(0.0%)	0	(0.0%)	5	(3.9%)	0	(0.0%)	5
Total	24	(100%)	37	(100%)	33	(100%)	127	(100%)	27	(100%)	248

Pairwise Comparisons: At least one disease grade is different from others ($p < 0.0001$)

Disease Stage	N	Missing Freq.	Mean	SD	Median	Min	Max	p-value
BPH	24	0	2.88	1.12	3	0	4	
PIN	37	3	4.54	1.45	5	0	6	
G6	33	1	5.06	1.12	5	3	7	<0.0001
G7/8/9/10	127	7	4.75	2.12	5	0	8	
AI/Meta	27	4	2.22	2.67	0	0	7	

Adjusted p-values of pairwise comparisons by Tukey's Method and Interpretation

- BPH is significantly lower than PIN, G6, and G7/8/9/10.
- AI/Meta is significantly lower than PIN, G6, and G7/8/9/10.
- No difference between AI/Meta and BPH. No difference between PIN, G6, and G7/8/9/10.

	PIN	G6	G7/8/9/10	AI/Meta
BPH	0.009	0.0003	0.0002	0.74
PIN		0.79	0.98	<.0001
G6			0.92	<.0001
G7/8/9/10				<.0001

Table S2. Depletion of specific tyrosine kinases causes a decrease in live PCSC cells.

Kinase	% Live CD44+ Cells	Kinase	% Live CD44+ Cells	Kinase	% Live CD44+ Cells
**ABL1**	107	**FES**	102	**MET**	129
**ABL2**	113	**FGFR1**	110	**MST1R**	120
**ALK**	82	**FGFR3**	104	**MSK**	127
**AXL**	94	**FGFR2**	96	**NTRK1**	110
**BLK**	117	**FGFR4**	103	**NTRK2**	109
**BMX**	104	**FGR**	102	**NTRK3**	115
**BTIC**	115	**FLT1**	90	**ROR1**	132
**DDR1**	95	**FLT3**	104	**ROR2**	100
**DDR2**	109	**FLT4**	93	**PDGFRA**	91
**PDGFRb**	110	**FRK**	99	**PTK2**	94
**CSK**	105	**FYN**	101	**PTK6**	102
**EGFR**	107	**HCK**	98	**PTK7**	92
**EPHA2**	107	**IGF1R**	103	**RET**	95
**EPHA1**	116	**INSR**	105	**ROS1**	111
**EPHA3**	105	**INSRR**	104	**RYK**	121
**EPHA4**	115	**ITK**	103	**SRC**	104
**EPHA5**	111	**JAK1**	105	**SRMS**	132
**EPHA7**	117	**JAK2**	104	**SYK**	105
**EPHA8**	108	**JAK3**	103	**TEC**	105
**EPHB1**	108	**KDR**	105	**TEK**	119
**EPHB2**	101	**KIT**	97	**TIE1**	103
**EPHB3**	107	**LMTK2**	105	**TNK1**	73
**EPHB4**	110	**LTK**	101	**ACK1**	**64**
**EPHB6**	102	**LYN**	105	**TXK**	99
**ERBB2**	96	**MATK**	104	**TYK2**	84
**ERBB3**	89	**Control**	100	**TYRO3**	101
**ERBB4**	107			**WEE1**	92
**PTK2B**	92			**YES1**	85
**FER**	104			**ZAP70**	110
**Control**	100			**Control**	100
